# Excess cerebral oxygen delivery follows return of spontaneous circulation in near-term asphyxiated lambs

**DOI:** 10.1038/s41598-020-73453-x

**Published:** 2020-10-05

**Authors:** Shiraz Badurdeen, Andrew W. Gill, Martin Kluckow, Calum T. Roberts, Robert Galinsky, Sarah Klink, Suzanne L. Miller, Peter G. Davis, Georg M. Schmölzer, Stuart B. Hooper, Graeme R. Polglase

**Affiliations:** 1 The Ritchie Centre, The Hudson Institute of Medical Research, 27-31 Wright St, Clayton, VIC 3168 Australia; 2 Newborn Research, Royal Women’s Hospital, Melbourne, VIC Australia; 3 Centre for Neonatal Research and Education, University of Western Australia, Perth, WA Australia; 4 Department of Neonatology, Royal North Shore Hospital, University of Sydney, Sydney, NSW Australia; 5 Department of Paediatrics, Monash University, Clayton, VIC Australia; 6 Monash Newborn, Monash Children’s Hospital, Clayton, VIC Australia; 7 Department of Pediatrics, University of Alberta, Edmonton, Canada; 8 Department of Obstetrics and Gynaecology, Monash University, Victoria, Australia

**Keywords:** Experimental models of disease, Paediatric research, Preclinical research, Translational research

## Abstract

Hypoxic-ischaemia renders the neonatal brain susceptible to early secondary injury from oxidative stress and impaired autoregulation. We aimed to describe cerebral oxygen kinetics and haemodynamics immediately following return of spontaneous circulation (ROSC) and evaluate non-invasive parameters to facilitate bedside monitoring. Near-term sheep fetuses [139 ± 2 (SD) days gestation, n = 16] were instrumented to measure carotid artery (CA) flow, pressure, right brachial arterial and jugular venous saturation (SaO_2_ and SvO_2_, respectively). Cerebral oxygenation (crSO_2_) was measured using near-infrared spectroscopy (NIRS). Following induction of severe asphyxia, lambs received cardiopulmonary resuscitation using 100% oxygen until ROSC, with oxygen subsequently weaned according to saturation nomograms as per current guidelines. We found that oxygen consumption did not rise following ROSC, but oxygen delivery was markedly elevated until 15 min after ROSC. CrSO_2_ and heart rate each correlated with oxygen delivery. SaO_2_ remained > 90% and was less useful for identifying trends in oxygen delivery. CrSO_2_ correlated inversely with cerebral fractional oxygen extraction. In conclusion, ROSC from perinatal asphyxia is characterised by excess oxygen delivery that is driven by rapid increases in cerebrovascular pressure, flow, and oxygen saturation, and may be monitored non-invasively. Further work to describe and limit injury mediated by oxygen toxicity following ROSC is warranted.

## Introduction

Hypoxic-ischaemic encephalopathy (HIE) continues to impose a 45–55% risk of death or moderate–severe disability despite the introduction of therapeutic hypothermia^1–3^. Management of infants with loss of cardiac output currently focuses on the rapid achievement of lung aeration, oxygenation and return of spontaneous circulation (ROSC). In the immediate period following ROSC, there are scarce data to inform a brain-protective stabilisation approach^4,5^.

One important consideration is the impact of oxygenation and supplemental oxygen provision on brain injury. Delivery room management aims to balance hypoxia–ischaemia duration with excessive oxygen delivery and oxidative stress^6–8^. Ensuring adequate oxygen delivery is vital for restoring aerobic metabolism and preserving brain tissue. However, commencing ventilation with room air results in lower mortality compared to using 100% fraction of inspired oxygen (FiO_2_)^9^. For infants receiving ventilatory support alone at birth, current guidelines recommend the adjustment of FiO_2_ to target oxygen saturation levels observed in transitioning normal term infants. In contrast, for infants who require cardiopulmonary resuscitation (CPR), guidelines recommend 100% FiO_2_ until ROSC is achieved^4,5^. Thereafter, FiO_2_ is titrated *down* according to oxygen saturation nomograms. There are no studies evaluating the impact of this approach on cerebral oxygen kinetics in the transitioning circulation^10^. As oxygenation is restored and aerobic respiration resumes, the kinetics of brain oxygen consumption is not known. We considered that brain oxygen consumption may rise in response to the accumulated oxygen deficit in keeping with mitochondrial studies of mild-moderate perinatal asphyxia^11,12^, or remain depressed from the antecedent asphyxia and ongoing suppression of cellular metabolic activity. Excessive oxygen exposure to the asphyxic neonatal brain is associated with free-radical mediated injury and apoptosis^13^.

Cerebral oxygen delivery is determined by the product of blood oxygen content and cerebral blood flow. In response to severe hypoxic-ischaemia, cardiac output is redistributed to prioritise oxygen delivery to the brain through dilation of the cerebral vasculature. Lamb experiments have previously shown a rapid increase in both cerebral blood flow and oxygen delivery following ROSC^14–17^. The adequacy of oxygen delivery however is related to oxygen consumption. While hypoxic-ischaemia clearly represents a gross mismatch between these two variables, the kinetics in the immediate period after ROSC are not known. A measure of the matching of delivery and consumption is the cerebral fractional oxygen extraction (cFOE). This is the proportion of oxygen unloaded from haemoglobin (Hb) into the tissue. cFOE may be derived from the Fick principle as the difference in oxygen content of blood entering and leaving the brain^18^. From a physiological standpoint, oxygen sufficiency may be defined as oxygen delivery that is well matched to oxygen consumption, and is seen in the relatively stable cFOE during normal transition^18–20^.

A second consideration is the consequence of impaired autoregulation on exposure of the cerebral microvasculature to pressure^8^. Uncoupling of autoregulation renders the cerebral circulation pressure-passive. Recent studies in asphyxic term-equivalent lambs showed an association between early cord clamping, rebound hypertension following ROSC, and markers of cerebral injury including blood vessel protein extravasation and loss of tight-junction integrity^16^. Blood pressure is not routinely measured in the delivery room and is limited by the interference of cuff inflation with pulse oximetry on the preductal arm^21^.

For monitoring of oxygen kinetics and cerebrovascular pressure to be useful in the delivery room, non-invasive measures must be identified. Pulse oximetry is routinely used for monitoring arterial oxygen saturation (SpO_2_) and heart rate (HR). Near Infrared Spectroscopy (NIRS) measures regional cerebral oxygenation (crSO_2_), which represents a mixed saturation that is largely determined by the venous compartment- the arterio-venous ratio ranges between 25:75 to 30:70^22^. As such, crSO_2_ may be used as a surrogate for venous oxygen saturation to calculate cerebral fractional tissue oxygen extraction (cFTOE) and thereby assess the balance of oxygen delivery and consumption^18,23,24^. The utility and validity of these non-invasive measures in the immediate period following ROSC in neonatal asphyxia has not been described.

Using a well-established newborn lamb model that imposes asphyxia on a transitioning circulation^16,25^, we aimed to:

(i) describe the changes in cerebral oxygen kinetics and cerebrovascular pressure following ROSC
(ii) identify periods of mismatch between oxygen delivery and oxygen consumption
(iii) identify non-invasive physiological correlates of oxygen delivery and cerebrovascular pressure.

We hypothesised that CPR with 100% FiO_2_ in an asphyxiated lamb model would result in an imbalance of cerebral oxygen kinetics in the immediate recovery period following ROSC.

## Methods

Experimental procedures were conducted in accordance with the National Health and Medical Research Council of Australia’s guidelines and were approved by the Monash University Animal Ethics Committee. Experiments are reported in compliance with the ARRIVE guidelines^26^.

### Instrumentation and delivery

Pregnant Border-Leicester ewes (*Ovis aries*) at 139 ± 2 days’ gestation (mean ± SD; term ~ 148 days) were housed in floor pens with access to food and water ad libitum and were monitored daily for health and wellbeing. All experiments were carried out between 7am—5 pm in a dedicated non-sterile animal laboratory operating facility. Ewes were anaesthetised by intravenous injection of thiopentone sodium (20 mg/ kg; Jurox, NSW, Australia), followed by tracheal intubation and delivery of inhaled anaesthesia (isoflurane 1.5%–2.5% in oxygenated air; Bomac Animal Health, NSW, Australia). The oxygen concentration of the carrier gas was titrated between 21–80% FiO_2_ to maintain SpO_2_ between 90–95%. The median (interquartile range) SpO_2_ of the ewes across all experiments was 93% (90–95%). The fetal head and chest were exposed via hysterotomy for placement of an ultrasonic flow transducer of appropriate size (Transonic Systems, Ithaca, NY, USA) around the carotid artery, and left main pulmonary artery accessed via a left thoracotomy. Pressure transducers (PD10; DTX Plus Transducer; Becton Dickinson, Singapore) were used to measure invasive blood pressure in the carotid and brachial arteries. Catheters were inserted into a jugular vein and right brachial artery for central venous and pre-ductal arterial blood gas sampling respectively. After closure of the incisions in the neck and chest, the fetal trachea was intubated with a 4.5 mm cuffed endotracheal tube and lung liquid was passively drained. A transcutaneous arterial oxygen saturation probe (Masimo SET Pulse Oximeter Sensor LNCS Neo-3, Irvine CA, USA) was placed around the right forelimb of the lamb and used to guide FiO_2_ in accordance with international guidelines^4,5^. A Near Infrared Spectroscopy optode (Casmed Foresight, CAS Medical Systems, Branford, CT, USA) was placed on the shaved scalp over the left frontal cortex, secured using opaque material and used to continuously measure cerebral tissue oxygen saturation. A rectal probe was inserted to monitor temperature.

Continuous digital recordings were made in real time (1 kHz) using a data acquisition system (PowerLab; ADInstruments, NSW, Australia) for the following parameters: instantaneous blood flows in the carotid artery and left pulmonary artery, carotid and brachial arterial pressures, airway pressures, tidal volumes, cerebral tissue and preductal oxygenation.

After completion of instrumentation, the fetus was completely exteriorised from the uterus, still attached to the umbilical cord, dried, and placed on a delivery table immediately next to the ewe. Physiological parameters were allowed to stabilise prior to the birth procedures. Asphyxia was induced by cord clamping or internal iliac artery occlusion without ventilation as previously described^16,27^. At the point of terminal fetal asphyxia, defined by asystole and mean arterial blood pressure of ~ 0 mmHg, ventilation was initiated with a 30 s sustained inflation at 30 cmH2O pressure with FiO_2_ of 100% (Babylog 8000+, Dräger, Lübeck, Germany). This was followed by positive pressure ventilation in volume-guarantee mode (7 mL/kg) with end-expiratory pressure of 5 cmH_2_O, inspiratory time 0.5 s, expiratory time 0.5 s and rate of 60 ventilations per minute for a total of 30 min. Lambs were ventilated with warm humidified air and sedated (Alfaxalone intravenous 5–15 mg/kg/h in 5% dextrose; Jurox) to prevent spontaneous breathing.

At one minute after the commencement of ventilation, manual chest compressions were started at a rate of approximately 90 compressions per minute. If required, adrenaline (1:10,000 0.2 ml/kg) was administered one minute after the initiation of chest compressions and at 3-min intervals until ROSC. Chest compressions and ventilations were continued until the lamb achieved ROSC, defined as heart rate greater than 100 beats per minute and mean blood pressure of > 30 mmHg.

Following ROSC, management was the same for all animals. FiO_2_ was adjusted from 100% to target arterial oxygen saturation between 88 and 95%. Ventilation was adjusted to maintain PaCO_2_ between 45 and 55 mmHg. Resuscitation measures including ventilator changes to achieve oxygen saturation and PaCO_2_ targets were performed by the same 2 neonatologists for all lambs (AG and MK).

Sequential blood samples were collected via the brachial artery catheter and blood gas parameters measured using a blood gas analyser (ABL90 Flex Plus, Radiometer, Copenhagen, Denmark). The first was taken before induction of asphyxia, then at terminal asphyxia, and then at 3, 6, 9, 12, 15, 20, 25 and 30 min after ROSC. Jugular venous blood samples were drawn simultaneous with arterial sampling at the following timepoints: before asphyxia, at terminal asphyxia, and 3, 9, 15, 25 and 30 min after ROSC. Lambs were then euthanised with sodium pentobarbitone (> 100 mg/kg intravenous), major organs excised and weighed.

### Sample size and data analysis

All lambs studied in a single experimental week were prospectively included for the current study (n = 16). LabChart recordings from each lamb were analysed by extracting 10 s intervals for each physiological parameter at timepoints that corresponded to blood gas sampling. Data were analysed using GraphPad Prism version 8 for Mac OS X (GraphPad Software, La Jolla California USA).

1 $$ {\text{Arterial oxygen content }} = \, \left( {\left[ {{1}.{39}*{\text{Hb}}*{\text{SaO}}_{{2}} /{1}00} \right] \, + \, \left[ {0.00{3}*{\text{PaO}}_{{2}} } \right]} \right), $$

where Hb is the haemoglobin concentration (g/dL). Cerebral oxygen delivery (ml/kg/min) = (Mean cerebral blood flow* Arterial oxygen content)/brain weight (kg).

2 $$ {\text{Cerebral oxygen consumption }}({\text{ml}}/{\text{kg}}/{\min}) \, = \, ({\text{Mean cerebral blood flow }} \times \, [{1}.{39}*{\text{Hb}}*({\text{SaO}}_{{2}} {-}{\text{ SvO}}_{{2}} )/{1}00])/{\text{brain weight (kg)}} $$

3 $$ {\text{Cerebral fractional tissue oxygen extraction from NIRS (cfTOE NIRS}}, \, \% {) } = \, [({\text{SaO}}_{{2}} {-}{\text{ crSO}}_{{2}} )/{\text{SaO}}_{{2}} ]*{1}00 $$

4 $$ {\text{Calculated cerebral fractional oxygen extraction (calculated cFOE}}, \, \% {) } = \, [({\text{SaO}}_{{2}} {-}{\text{ SvO}}_{{2}} )/{\text{SaO}}_{{2}} ]*{1}00 $$

5 $$ \begin{gathered} {\text{Calculated crSO}}_{{2}} \left( \% \right) \hfill \\ \quad {\text{at arterio}} - {\text{venous ratio of 3}}0:{7}0 \, = \, 0.{30}*{\text{SaO}}_{{2}} + \, 0.{70}*{\text{SvO}}_{{2}} \hfill \\ \quad {\text{at arterio}} - {\text{venous ratio of 2}}5:75 \, = \, 0.25*{\text{SaO}}_{{2}} + \, 0.75*{\text{SvO}}_{{2}} \hfill \\ \quad {\text{at arterio}} - {\text{venous ratio of 15}}:{85 } = \, 0.15*{\text{SaO}}_{{2}} + \, 0.{85}*{\text{SvO}}_{{2}} \hfill \\ \end{gathered} $$

### Statistical analysis

Cardiorespiratory parameters at each timepoint after ROSC were compared to fetal levels using one-way repeated measures analysis of variance. Bonferroni correction with a single pooled variance was used for multiple comparisons. Where there was missing data, a mixed effects model was used. Analysis was performed using PRISM version 8 for Mac OS X (GraphPad Software, La Jolla California USA).

To assess associations between measured parameters accounting for the variation between subjects, we calculated within-subject correlations for repeated measures using the rmcorr package in *R* version 3.6.0^28–30^. Agreement between parameters was assessed with Bland Altman plots, adjusting for multiple measurements per lamb under changing conditions (‘true value varies’)^31^, using MedCalc for Windows, version 15.0 (MedCalc Software, Ostend, Belgium).

## Results

Four lambs did not achieve ROSC. No changes to the experimental protocols were made. Data from all lambs that achieved ROSC (n = 12, mean weight 4.5 kg, standard deviation 0.8 kg) were combined for the present analysis. The mean (standard deviation) duration between onset of asphyxia and asystole was 19 min (9 min). The mean (standard deviation) duration between commencement of ventilation and ROSC was 4 min (1 min).

### Cerebral oxygen kinetics and cerebrovascular pressure following ROSC

**Figure 1:**
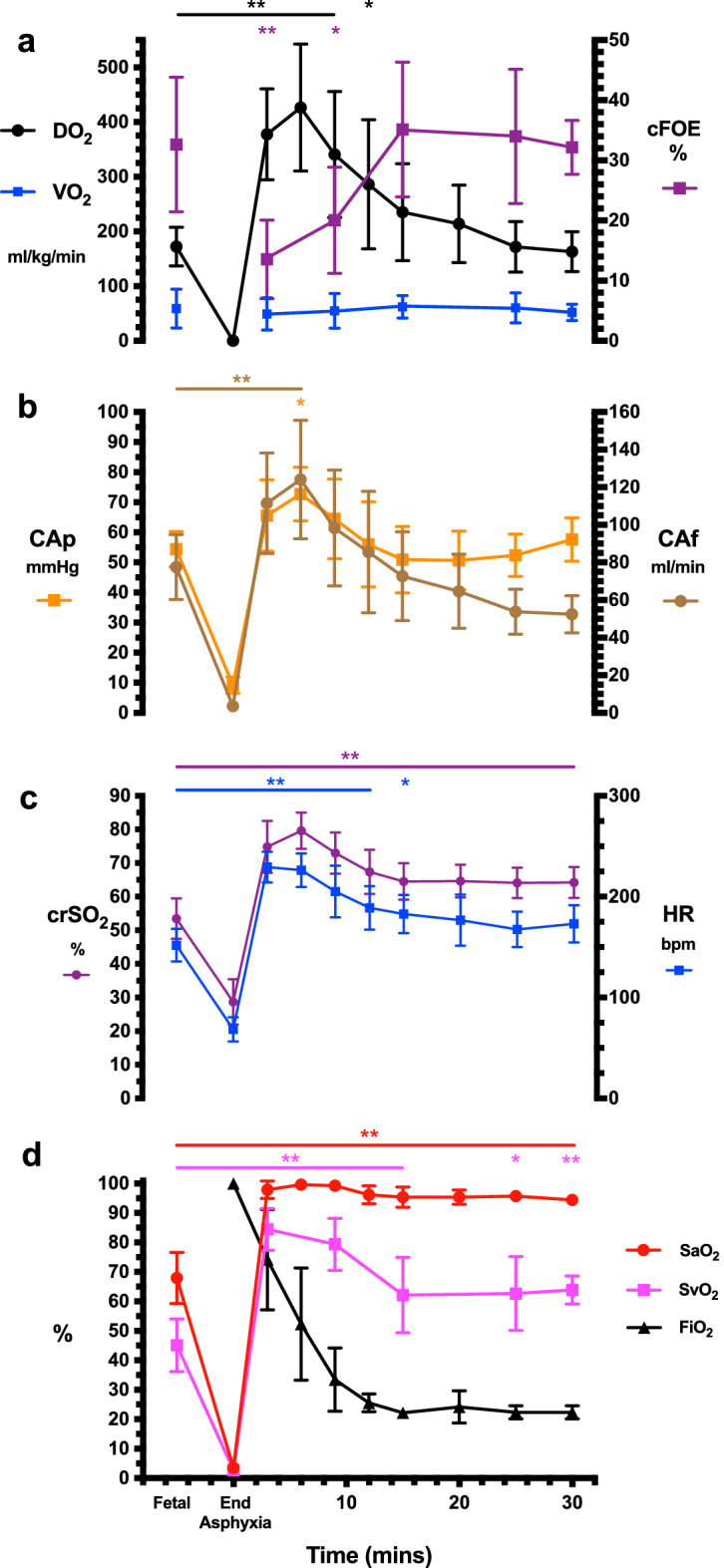
Changes in oxygenation and haemodynamic parameters following return of spontaneous circulation (ROSC) in asphyxiated near-term lambs showing **(a)** oxygen delivery (DO_2_), oxygen consumption (VO_2_) and cerebral fractional oxygen extraction (cFOE), **(b)** mean carotid artery blood pressure (CAp) and carotid artery blood flow (CAf), **(c)** regional cerebral oxygenation (crSO_2_) and heart rate (HR), **(d)** arterial oxygen saturation (SaO_2_), jugular venous oxygen saturation (SvO_2_) and fraction of inspired oxygen (FiO_2_). Means + /− 95% confidence interval of the mean are shown. Comparison is made to fetal levels for each timepoint after ROSC, with ** representing p < 0.01 and * representing p < 0.05.

Compared to fetal levels, cerebral oxygen delivery increased by a mean of 250 ml/kg/min at 6 min after ROSC (95% Confidence Interval 140–360 ml/kg/min, p < 0.0001), before returning to near fetal levels by 15 min (Fig. 1a). Cerebral oxygen consumption, reflecting metabolic demand, showed no change from fetal levels at all timepoints following ROSC. Accordingly, cerebral fractional oxygen extraction (cFOE) fell by a mean of 20% (95% Confidence Interval 7.0–32%, p < 0.001) at 3 min after ROSC and remained below fetal levels at 9 min. Thereafter, cFOE returned to near fetal levels, corresponding with the fall in oxygen delivery.

Both carotid artery flow and mean arterial pressure followed a similar pattern to oxygen delivery, rising by a mean of 44.6 ml/min and 18.3 mmHg respectively during the first 6 min after ROSC when compared to fetal levels (95% Confidence Intervals 15.5–73.7 ml/min, p < 0.001 and 2.6–34.1 mmHg, p = 0.01 respectively, Fig. 1b). This was followed by a return to near fetal levels by 9 min. All three parameters continued to track one another closely until 15 min after ROSC. Thereafter, carotid artery pressure trended upwards but was not matched by a rise in carotid artery flow or oxygen delivery (Fig. 1).

**Figure 2:**
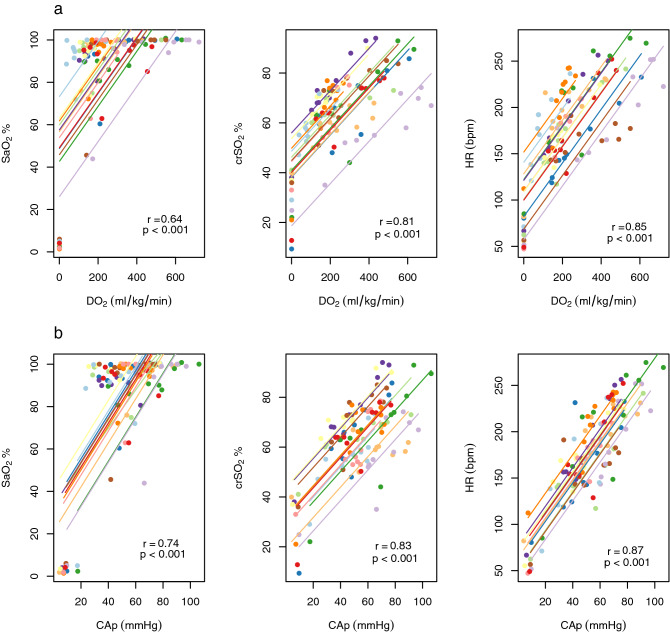
Correlations between non-invasive parameters and **(a)** oxygen delivery (DO_2_), **(b)** mean carotid arterial pressure (CAp). Longitudinal measurements and the correlation trend line are coloured per individual lamb.

The pattern of fluctuation in oxygen delivery, carotid artery flow and pressure were similar to changes in the non-invasive parameters of HR (mean HR 167–229 across timepoints) and crSO_2_ (mean crSO_2_ 64–80% across timepoints), but not SaO_2_. SaO_2_ increased sharply after ROSC and remained elevated (mean SaO_2_ 94%-100%) despite step-wise reductions in FiO_2_ (Fig. 1c,d). In keeping with these observations, HR and crSO_2_ correlated more closely than SaO_2_ with oxygen delivery and carotid artery pressure (Fig. 2).

**Figure 3:**
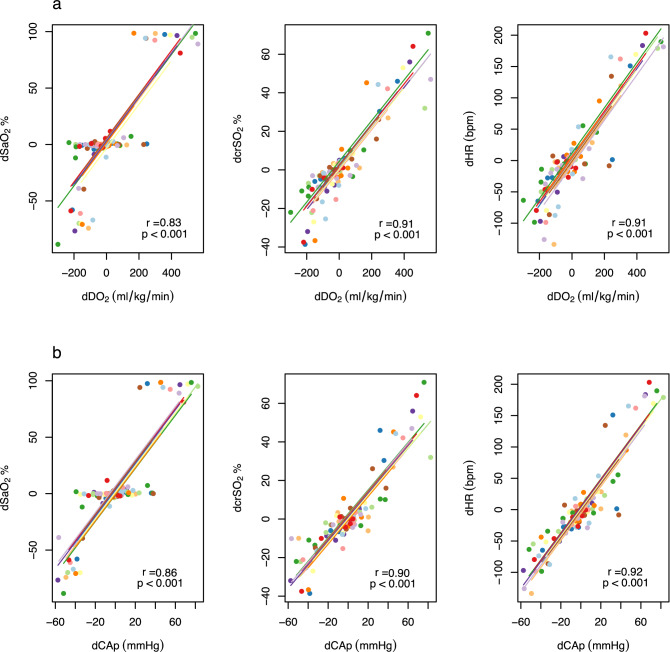
Correlations between changes in non-invasive parameters and changes in **(a)** oxygen delivery (DO_2_), **(b)** mean carotid arterial pressure (CAp) at adjacent timepoints. Longitudinal measurements and the correlation trend line are coloured per individual lamb.

We evaluated whether the change in HR, crSO_2_ and SaO_2_ between adjacent timepoints corresponded with fluctuations in oxygen delivery and carotid artery pressure. Changes in HR and crSO_2_ each correlated strongly with changes in both oxygen delivery and carotid artery pressure. SaO_2_ levels showed little change when oxygen delivery varied between − 100 to + 100 ml/kg/min, or when carotid artery pressure varied between − 25 to + 25 mmHg. An increase in HR by 80 beats per minute or crSO_2_ by 15% between timepoints corresponded in all instances with a rise in oxygen delivery by 150 ml/kg/min and/or an increase in carotid artery pressure by 20 mmHg (Fig. 3).

**Figure 4:**
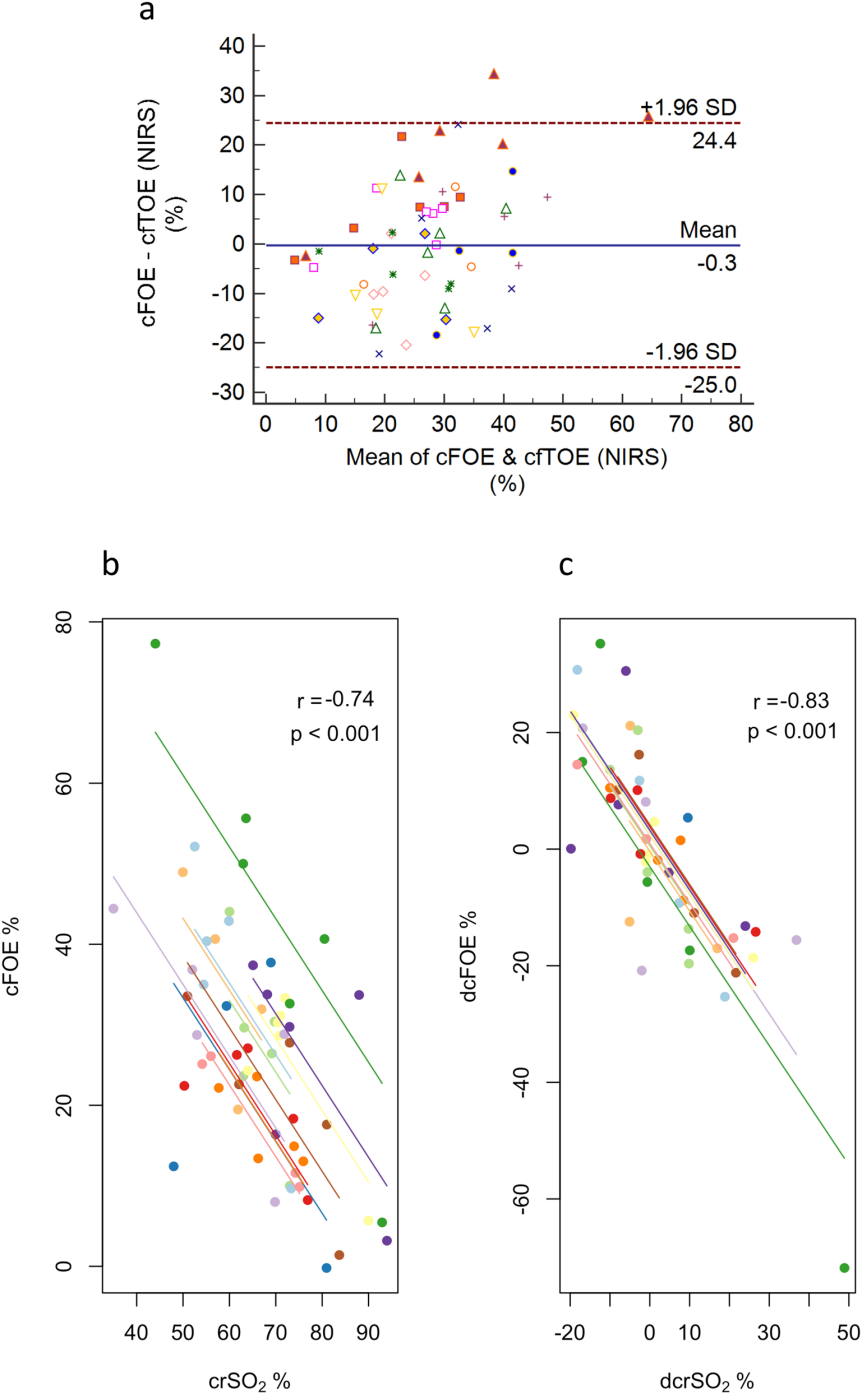
**(a)** Bland–Altman plot of cerebral fractional oxygen extraction (cFOE) as estimated using Near Infrared Spectroscopy (cfTOE NIRS) versus calculation from direct blood gas analysis. **(b)** Correlation between regional cerebral oxygenation from NIRS (crSO_2_) and cFOE calculated using blood gases, and **(c)** correlation between the changes in these two parameters at adjacent timepoints. Longitudinal measurements and the correlation trend line are coloured per individual lamb.

We next evaluated the utility of NIRS for estimating cerebral fractional oxygen extraction. First, we assessed the degree of agreement of tissue oxygen extraction calculated using crSO_2_ (cfTOE NIRS, Eq. 3) versus tissue oxygen extraction that is calculated using blood gases (calculated cFOE, Eq. 4). Figure 4a shows moderate agreement between these 2 parameters (95% CI -24.6 to 25.6). There was minimal bias (0.5%) suggesting little systematic deviation between SvO_2_ on the one hand and crSO_2_ from the arterial saturation contribution of the NIRS-derived value.

In the context of stable (high) SaO_2_, we asked whether a rise in crSO_2_ might reflect a drop in cFOE, indicating excessive oxygen delivery in relation to consumption. We found an inverse relationship between absolute values of crSO_2_ and cFOE as well as change in these parameters between adjacent timepoints (Fig. 4b,c).

**Figure 5:**
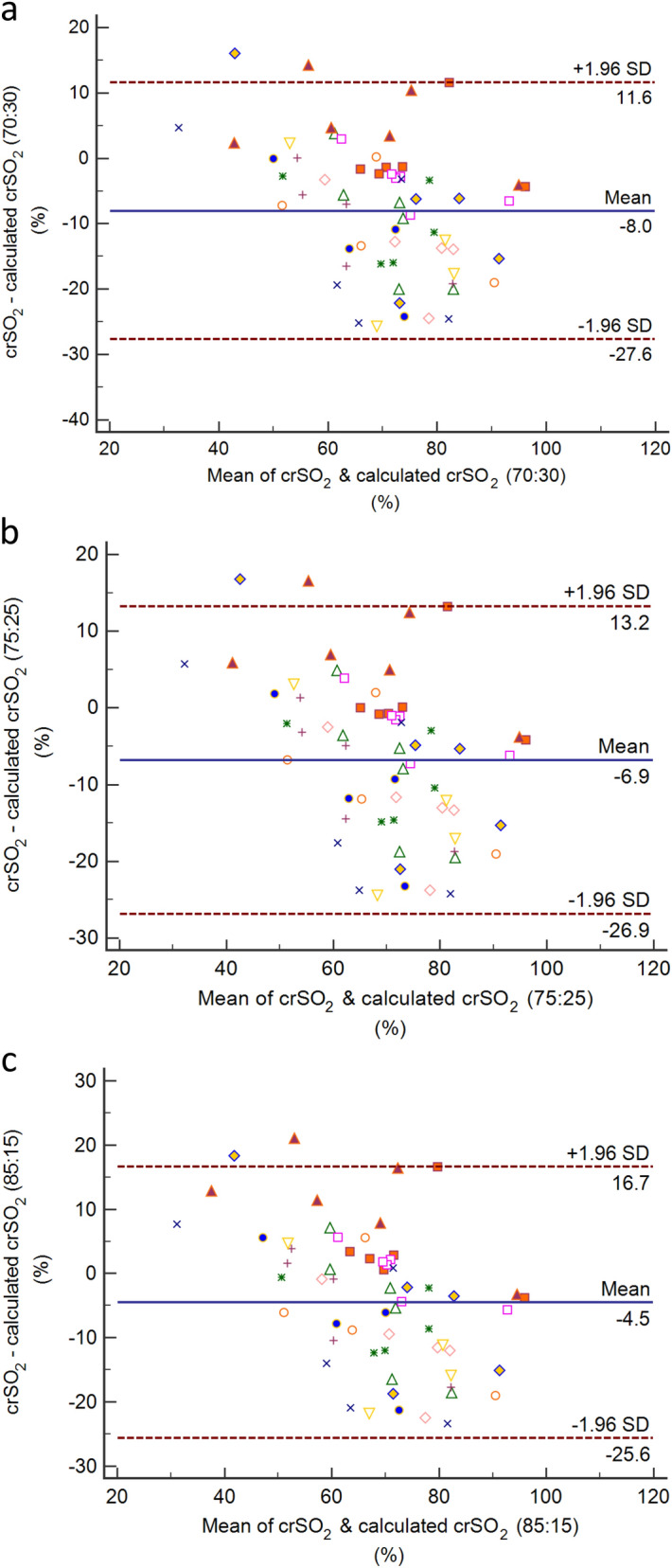
Bland–Altman plots of cerebral regional oxygenation (crSO_2_) as measured using NIRS versus crSO_2_ calculated using blood gas analysis at arteriovenous ratios of **(a)** 30:70, **(b)** 25:75 and **(c)** 15:85.

Given the findings in Fig. 4a, we explored the relative arterial and venous contributions to crSO_2_. We assessed the degree of agreement between crSO_2_ derived from NIRS, and crSO_2_ calculated from blood gases using different arterio-venous ratios (Eq. 5). As shown in Fig. 5, the manufacturer-reported arterio-venous ratio of 30:70 had a large bias (− 7.5%). This suggests that the NIRS-derived crSO_2_ during recovery from asphyxia is weighted more towards the venous compartment, with an arterio-venous ratio of 15:85 giving only a small bias (− 3.8%) and 95% CI of − 25.2 to 17.5. Additionally, there appeared to be an inverse relationship between crSO_2_ and bias, suggesting greater weighting towards the arterial compartment at lower levels of crSO_2_, or representing a delay in change in crSO_2_ values derived from NIRS in the context of rapidly changing saturation levels.

## Discussion

In this study we show that the immediate period following ROSC is characterised by an excess in oxygen delivery relative to consumption, driven by rapid increases in cerebrovascular pressure, flow, and oxygen saturation. We also show that fluctuations in oxygen delivery, fractional oxygen extraction and cerebrovascular pressure may be monitored using NIRS and HR while SpO_2_ levels remain > 90%.

Our findings of a rapid, transient increase in carotid artery flow and oxygen delivery following ROSC are consistent with previous lamb studies in the transitional period^14–17^. Studies in asphyxiated lambs and piglets receiving 100% FiO_2_ found higher cerebral tissue levels of oxygen than those in lambs that received 21% FiO_2_^14,32^. We found that the increase in oxygen delivery for lambs resuscitated in 100% FiO_2_ was not matched by an increase in oxygen consumption following ROSC to compensate for an accumulated oxygen deficit. In contrast to normal conditions where oxygen delivery in a newborn is approximately 3 times higher than consumption, as corroborated at the fetal and post-hyperaemic time points, we found oxygen delivery to be up to 8 times higher than consumption in the 15 min after ROSC^33^. Our findings are consistent with those of neonatal calf and lamb studies that were performed days after birth, well after the transitional period had occurred^34,35^.

We further demonstrate that the pattern of change in carotid artery flow (and consequently oxygen delivery) closely matches the fluctuation in CA pressure in the immediate period following ROSC, with subsequent decoupling between 20–30 min. Impaired cerebral autoregulation and hyperaemia occur in response to hypoxia, hypercapnia and metabolic derangement, rendering the cerebral circulation passive to the catecholamine-driven pressure surge^36–38^. Rebound hypertension following ROSC is associated with blood vessel protein extravasation and loss of tight-junction integrity in the subcortical and periventricular white matter and cortical grey matter^16^. Our findings suggest that the first 15 min after ROSC represents a critical period for impaired autoregulation and hypertension-mediated cerebrovascular injury.

The inadequacy of SaO_2_ monitoring for oxygen targeting is particularly apparent in the present context, where blood oxygen content changes little in comparison with cerebral blood flow. The latter is strongly dependent on cardiac output, which in the newborn is strongly modified by HR^39^. In keeping with this, both the trends in, and absolute values of HR correlated more strongly with oxygen delivery than SaO_2_. With minimal change in oxygen consumption following ROSC, excess oxygen delivered to the brain is carried over into the venous compartment. crSO_2_, and particularly trends in crSO_2_, correlated well with oxygen delivery and cFOE, reflecting the fact that crSO_2_ predominantly measures venous saturation. Given that cFOE indicates the degree of mismatch between delivery and consumption, our finding of an inverse relationship between trends in cFOE and crSO_2_ provides an opportunity for tailored oxygen therapy in the immediate period following ROSC.

CfTOE nomograms for the immediate transition period in normal term newborns have been described, showing initial high extraction, between 15–45% (25th–75th percentiles) at 3 min, that subsequently stabilises at between 10–25% (25th–75th percentiles) between 6–20 min after birth^20^. We found that this pattern is reversed in the post-ROSC phase, with high oxygen delivery and low cerebral metabolic rate resulting in low cFOE, until the return of autoregulatory control of cerebral blood flow and reduction in FiO_2_. Avoidance of cerebral oxygen excess or deficit may therefore be better achieved by targeting the cFOE nomograms of normal term infants (via targeting a gradual rise in crSO_2_) than the current practice of following SpO_2_ nomograms^18–20^. Targeting oxygen delivery based on cFOE or central venous oxygen saturation is commonly practiced in intensive care and anaesthetic management^40–42^, but is not routinely done in the delivery room.

In attempting to further define the NIRS-derived crSO_2_ measurement during the immediate period following ROSC, we found greater contribution of the venous compartment than the normally accepted ratio. This difference may vary with other NIRS devices and sensors. Manufacturer algorithms for calculating cerebral regional oxygen saturation vary and the differences in values derived from different devices may be oxygenation dependent^43,44^. Whilst the arterio-venous ratio we report is nearly identical to findings in children with congenital heart disease, the use of jugular venous oxygen saturation for central venous oxygen saturation that reflects both cerebral and extracerebral venous blood may be a contributory factor^45^. If oxygen extraction by extracerebral tissue is lower than cerebral tissue this would artificially increase central venous oxygen saturation and the venous contribution to the calculated arterio-venous ratio. An additional factor is the alterations in cerebral vasoreactivity at different levels of hypoxia. In keeping with our findings, a previous lamb study showed significant arterial vasodilatation at lower oxygenation, making the assumed arterio-venous ratio and the corresponding estimate of calculated crSO_2_ erroneously low^24^.

Our study has several limitations. The use of anaesthesia may have suppressed the cerebral metabolic rate. Although this would lead to an underestimation of cFOE immediately following ROSC, our findings are in keeping with other studies that found a marked surplus of oxygen delivery in relation to consumption following hypoxia induced outside the transitional period^34,35^. Studies of infants resuscitated following perinatal hypoxic-ischaemia are required to determine whether a similar pattern of change in crSO_2_ and/or cfTOE are observed in the delivery room. Secondly, we used jugular venous saturations rather than those of sagittal sinus blood. The former includes venous blood from extracerebral tissue and may overestimate the actual cerebral venous saturation^24,45^. However, validation studies of NIRS devices predominantly use human jugular bulb oxygen saturation measurements^46–48^. We did not directly measure brain tissue oxygen tension; the oxygen dissociation curve is likely to be shifted at the tissue level by the acidotic brain pH and other local factors. Finally, while it is well established that commencing respiratory support in high oxygen concentration for mildly asphyxic animals and human infants contributes towards neuronal excitotoxicity, free-radical-mediated injury and increased mortality, we do not provide direct evidence of pathological injury in this study of severe asphyxia. Further work with carefully designed experiments is required to establish whether such injury can be detected over and above the injurious effect of severe asphyxia, as well as whether prevention of excess oxygenation after ROSC is protective. Hyperoxic injury following adult cardiac arrest is well established and is currently the subject of randomised trials^49–51^.

In contrast to most previous studies of induced hypoxia, we studied oxygen kinetics and haemodynamics in the immediate neonatal transitional period, i.e. in the setting of a patent ductus arteriosus and high pulmonary vascular resistance. Following ROSC, the management of all lambs with respect to SaO_2_ and PaCO_2_ targets was performed simultaneously by the same 2 neonatologists (MK and AG) using a standardised approach and weaning of FiO_2_ to target SaO_2_. Our findings suggest that a review of current guidelines recommending SpO_2_ targeting during the resuscitation period following exposure to hypoxic-ischaemia is timely. They highlight a need to explore strategies to avoid excessive oxygen delivery. One strategy may be to use a lower FiO_2_ during CPR. The approach of using 100% FiO_2_ is based on the principle that cardiac muscle, which is grossly oxygen depleted to the point of arrest, may be reinvigorated more quickly by providing supplementary oxygen in addition to cardiac massage (which provides coronary flow). Animal studies, however, have consistently demonstrated that CPR in 21% oxygen is as efficacious in achieving ROSC as CPR in 100% oxygen^10,15^. While high levels of alveolar partial pressure of oxygen may be achieved during CPR in 100% FiO_2_, pulmonary flow is low despite chest compressions, so blood oxygen content is likely to be low^17^. Similarly, antegrade carotid flow is limited to the compression phase and oxygen delivery to the brain is likely to remain low until ROSC^17^. Maximising blood oxygen content during the low-output state that characterises the need for CPR may shorten the duration of severe cerebral hypoxia. On the other hand, use of 100% FiO_2_ during CPR may compound the burden of hyperoxia that we report here in the immediate post-ROSC phase. The relative contribution of hyperoxia during CPR versus the immediate post-ROSC period is not known and should be the subject of further investigation.

To date, no studies have investigated the efficacy of intermediate concentrations of oxygen for achieving either ROSC or for avoiding excessive oxygen delivery in the immediate post-ROSC phase. A second strategy would be to rapidly reduce FiO_2_ following the detection of ROSC, as is being trialed in adults^51,52^. In our study, the average FiO_2_ at 3, 6, 9 and 12 min after ROSC were 74%, 52%, 33% and 25% respectively. These timepoints corresponded to the highest period of oxygen exposure that may be mitigated by a more rapid weaning of FiO_2_ that is guided individually based on changes in crSO_2_ and/or HR. Consideration should also be given to the systemic effects of using different concentrations of oxygen. In particular, supplemental oxygen has an additive effect to lung aeration for reducing pulmonary vascular resistance after birth and is commonly necessary to achieve transition in preterm infants^53,54^. However, most full-term infants with perinatal asphyxia have compliant lungs at birth. It is likely that the necessary increase in pulmonary blood flow can be achieved while minimising supplemental oxygen use and risk of hyperoxic injury^17,55,56^. This should be the subject of further investigation in a transitional model of severe asphyxia like the one we report here.

In this study we report hyperoxia relative to consumption following complete cardiac arrest. Oxygen kinetics may be different with milder states of asphyxia, for example where adequate cardiac output is restored with respiratory support and/or a brief period of chest compressions. However, we and others have previously reported that the sympathetically-driven rebound increase in carotid blood pressure and flow occurs both in milder states of asphyxia, including when neither chest compressions nor adrenaline are used, as well as in brief repeated asphyxia^16,38,57^. This suggests that the immediate recovery phase in milder states of asphyxia is similarly susceptible to hyperoxic injury from supplemental oxygen, and may be the basis of increased mortality seen in randomised trials of newborns commencing respiratory support in 100% FiO_2_ compared to room air^9^.

In summary, we demonstrate that the first 15 min following ROSC from perinatal asphyxia is characterised by excess oxygen delivery relative to consumption when CPR is performed in 100% FiO_2_. Changes in cfTOE and HR may facilitate closer monitoring of fluctuations in oxygen delivery, cFOE and carotid artery pressure while SpO_2_ levels remain > 90%. Further work to describe injury mediated by oxygen toxicity following ROSC, and the role of physiologically-targeted resuscitation to mitigate such injury, would be timely.

## Data Availability

The datasets generated during and/or analysed during the current study are available from the corresponding author on reasonable request.
